# Measurement of salivary testosterone in adolescents and young men with Duchenne muscular dystrophy

**DOI:** 10.1186/s12902-021-00727-4

**Published:** 2021-04-10

**Authors:** Yolanda Alins Sahun, Tim Cheetham, Christopher Boot, Volker Straub, Claire L. Wood

**Affiliations:** 1 Plymouth Hospitals NHS Trust. Department of Paediatrics, Plymouth, UK; 2 Royal Victoria Infirmary. Department of Paediatric Endocrinology, Newcastle upon Tyne, UK; 3 Institute of Translational and Clinical Research, Faculty of Medical Sciences, Newcastle University, Newcastle upon Tyne, UK; 4 Royal Victoria Infirmary. Department of Clinical Biochemistry, Newcastle upon Tyne, UK; 5 The John Walton Muscular Dystrophy Research Centre and MRC Centre for Neuromuscular Diseases, Institute of Genetic Medicine, Newcastle upon Tyne, UK

**Keywords:** Salivary testosterone, Duchenne muscular dystrophy, Androgen deficiency, Long-term glucocorticoid therapy

## Abstract

**Background:**

Many young adults with Duchenne Muscular Dystrophy (DMD) receive long-term glucocorticoids (GC). GC can cause hypogonadotrophic hypogonadism and adolescents may therefore be candidates for pubertal induction. It is unclear whether men with DMD on or off GC have age-appropriate endogenous testosterone production.

**Methods:**

We undertook a quality improvement project to assess the feasibility of measuring salivary testosterone (SalT) levels in men with DMD at home. A Sal-T sampling kit was sent by post to all patients with DMD, aged 17 and older, registered at the John Walton Muscular Centre in Newcastle (*n* = 75). Submitted Sal-T samples were collected and submitted for analysis.

**Results:**

Twenty-eight out of seventy-five patients returned samples (age range: 17–34 years). 6/28 samples were unsuitable for analysis. Overall Sal-T levels (*n* = 22) were significantly lower than in the healthy population (178 ± 107 v 287 ± 109 pmol/l, *p* = 0.0001). Sal-T was lower in those on GC compared to those off GC (144 ± 81 versus 218 ± 125 pmol/l, *p* = 0.05). Three patients were unable to collect a sample due to ventilator dependence.

**Conclusion:**

Sal T can provide information about androgen status in DMD patients at home, overcoming barriers such as mobility difficulties and challenging venepuncture. However we only obtained samples in a minority of patients suggesting that Sal-T measurement may not be appropriate or acceptable to everyone. There needs to be a more detailed exploration of the barriers to sample submission.

**Supplementary Information:**

The online version contains supplementary material available at 10.1186/s12902-021-00727-4.

## Background

The improving health of adolescents and young adults with Duchenne muscular dystrophy (DMD) has resulted in changing expectations with many seeking to establish relationships and to lead independent adult lives. The majority of young people with DMD in the UK receive long-term, high dose glucocorticoid (GC) therapy, consistent with the published standards of care [1]. GC in pharmacological doses delay or inhibit activation of the hypothalamo-pituitary gonadal axis in adolescence with associated hypogonadotrophic hypogonadism and androgen deficiency. Androgen deficiency is frequently managed with exogenous testosterone replacement in order to promote virilisation and the development of muscle and bone mass. Low testosterone levels are associated with low mood and treatment of hypogonadism in young men is associated with improved well-being.

We routinely discuss the potential benefits of pubertal induction with testosterone (T) in adolescents with DMD [2]. However, the androgen status of older individuals with DMD, on or off long-term GC and irrespective of whether they have received earlier androgen therapy is unknown. Few adolescents continue with T supplementation through into adulthood in our experience and there is a cohort of older men with DMD whose androgen status is unknown.

Assessing androgen status in this population is challenging. Adults with DMD often have difficult venous access and don’t routinely have venepuncture for other reasons. Furthermore, for mobility reasons, patients often attend outreach clinics in the afternoon and so it is a logistical challenge to obtain a testosterone level in the morning when it is most informative.

Salivary testosterone (Sal-T) assays using liquid chromatography tandem-mass spectrometry (LC-MS/MS) are validated [3] and age-adjusted reference levels for the adult population are available [4]. Sal-T measurements potentially provide a non-invasive, stress-free method of capturing androgen status in this population at home, without the burden of attending a hospital appointment. Testosterone circulating in the body readily diffuses across capillaries and salivary ducts, resulting in a salivary portion that contains free, unbound testosterone which is not affected by salivary flow rates. Testosterone in saliva is thought to be closely related to the component of circulating T to which tissue androgen receptors are exposed, and a recent study showed a good correlation (r = 0.71) between Sal-T and free serum T levels in men [3]. Unlike serum T however, Sal-T levels are not affected by variations in sex hormone binding globulin and albumin.

## Methods

**Fig. 1:**
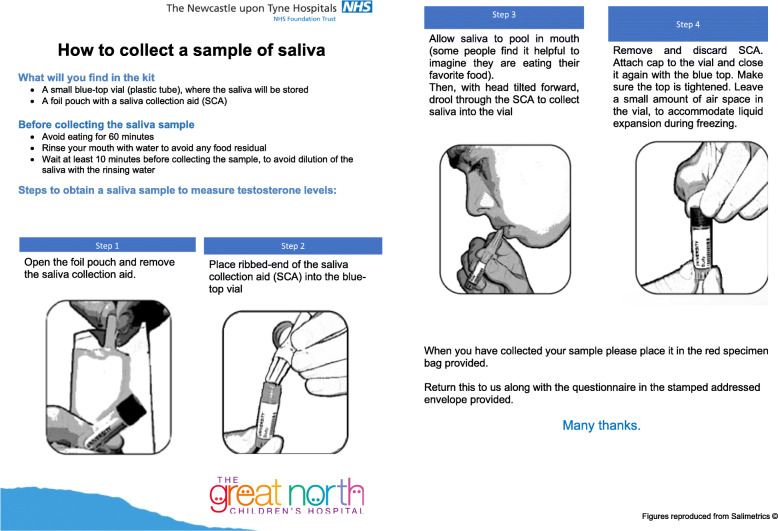
The information sheet sent to participants to describe how to collection a sample of saliva

We undertook a quality improvement project (QI) to assess the feasibility of measuring Sal-T levels in men with DMD without the need to attend hospital. We were particularly keen to assess the androgen status of patients with ongoing GC exposure. A Sal-T sampling kit (Salimetrics®, Carlsbad, CA92008) with instructions showing how to collect a saliva sample was sent by post to all patients with DMD, aged 17 and older, registered at the John Walton Muscular Centre in Newcastle (*n* = 75), see Fig. 1.

Briefly, participants were asked to drool down a plastic straw into a collection vial. To avoid assay interference/ contamination with blood, participants were asked to:

- Avoid eating for 60 min
- Rinse mouth with water to avoid any food residual
- Wait at least 10 min before collecting the sample, to avoid dilution of the saliva with the rinsing water

A short questionnaire was also included (see Supplementary data 1). Within this, participants were asked:

- Name and date of birth
- Date and time of sample collection
- Pubertal staging (using Tanner self-assessment pictures)
- Details of current or previous testosterone and/or glucocorticoid usage

A follow-up telephone call was made 2–4 weeks later to address any outstanding questions and encourage sample return.

After discussion with the Ethics Committee chair of NRES Committee North East- Newcastle and North Tyneside, it was concluded that ethical approval was not required for this study as it was considered a QI project (registered with Newcastle upon Tyne Hospitals Foundation Trust, project number 10158) that was intended to assess androgen status using an established technique. Assessing androgen status is regarded as being an important component of good clinical care. Consent for publication was therefore not obtained from patients, with only anonymised data included in the publication.

Sal-T levels were collected at participant’s homes and sent by first class post to the laboratory in Newcastle, where they were sent on the day of arrival, at ambient temperature, to South Manchester University Hospital for batch analysis using LC-MS/MS (the same assay platform from which the reference ranges are derived). Samples were stored at − 80 °C until analysis. A detailed methodology has already been published [3]. Intra- and inter assay CV’s were < 13% at 4 different concentrations analysed. Previous sample stability studies have shown no appreciable loss in T over a 5-day period [3], thus making it feasible for participants to produce the sample at home and post it to the laboratory.

Salivary testosterone data was checked for normality and t-tests used to compare the mean Sal-T level to the reference population and evaluate by GC status. Mean ± 1 standard deviation are shown. Statistical analysis was performed using Stata v15. A *p*-value of < 0.05 was considered statistically significant.

The patient and family doctor were informed of the outcome of Sal-T measurement, highlighting whether this was within the reference range or not.

## Results

Twenty-eight out of seventy-five patients returned samples by post to our Paediatric Endocrinology Service (age range: 17–34 years). Twenty two were suitable for analysis (one mislaid, four insufficient and in one instance the kit was inadvertently sent to someone who was too young). Three patients were unable to collect a sample due to poor health with associated ventilator dependence. 17/22 samples were collected after 10 am.

**Fig. 2:**
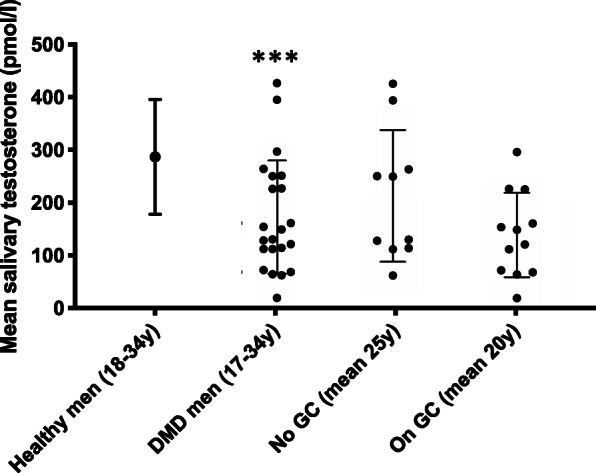
Mean salivary testosterone levels (+/−1SD) in Duchenne muscular dystrophy (DMD) patients both on and off glucocorticoids (GC), showing lower levels in those on GC and a significant reduction compared to the healthy male population of the same age (*** signifies *p* < 0.001 when comparing DMD men to healthy population)

Figure 2 shows that Sal-T levels in this cohort were significantly lower than data from a healthy male population of the same age group (178 ± 107 v 287 ± 109 pmol/l, *p* = 0.0001) [4] despite 6 patients (17 to 23 years) already being on testosterone supplementation. Sal-T was lower in the 12 patients on GC compared to the 10 patients who were not receiving GC (144 ± 81 versus 218 ± 125 pmol/l, *p* = 0.05). The mean age of the patients on GC was lower than those not on GC (20 ± 2 years v 25 ± 2 years for those off GC, *p* = 0.007). In the GC-treated group who had undergone pubertal induction with testosterone, Sal-T was highest in those currently receiving testosterone (6 participants) compared to those who had finished their treatment (209.5 versus 99 pmol/l). Levels were lowest in those never exposed to testosterone who were Tanner stage 1 and 2 (mean 77 pmol/l with 2 samples below lower limit of normal range).

## Discussion

This project has provided information about the androgen status of young adults with a rare disease, DMD, as well as providing information about the feasibility of assessing androgen status by measuring salivary testosterone in the home environment. Although the sample size was small, DMD is a rare disease and we believe that this cohort of young men represents one of the largest at any centre in the UK. This project suggests that Sal-T can be used to screen and monitor androgen status in some but not all patients with DMD. Although we contacted a relatively large number of young people with DMD we were only able to obtain data on a minority of patients. The relatively low response (37% of samples returned) may reflect the complexity of our DMD population including a wide age range, varying degrees of disability and issues such as moving address. The 3 patients who were unable to send a sample represent those with the worst pulmonary function. Older, sicker patients and those in residential care appeared to be less able or willing to submit samples. The fact that 14% of the samples sent were insufficient highlights the importance of using the correct technique to ensure that enough saliva is obtained, that contamination with blood is avoided and that sufficient time has elapsed since mouth hygiene.

Ideally samples should be taken when subjects wake up in the morning but we did not specify a time when the sample should be collected. Our emphasis in this exploratory study was on optimising the number of samples submitted. We did not want to pressurise young men into taking samples at a particular time and most were collected after 10 am which we suspect reflects their home routine (17/22). Analysis of diurnal variation in initial studies demonstrated a significant reduction in salivary testosterone levels when samples were taken in the afternoon compared to before 10 am [3]. The 5 morning samples in our study were dispersed evenly throughout the groups and re-running the GC analysis excluding those samples did not alter the results.

The salivary testosterone levels in patients with DMD on GC were lower than those off-GC and compared to age-matched reference data, even though some of the patients on GC were already on testosterone supplementation. Patients on GC were younger and it is unclear the extent to which GC prevent or simply delay progress through puberty in this patient group. What impact testosterone supplementation will have on the physical and psychological well-being of adult patients on GC therapy is also unknown.

## Conclusions

To our knowledge this is the first attempt to use home Sal-T sampling as a means of improving patient care. Our desire was to reduce inconvenience and cost for a group of patients with complex, chronic health needs. Before this technique can be used more widely there needs to be a better understanding of the implications of testosterone status in these adolescents and young men and a more detailed exploration of the barriers to sample submission and analysis.

## Supplementary Information

**Additional file 1.** Self-assessment questionnaire.

## Data Availability

The datasets used and/or analysed during the current study are available from the corresponding author on reasonable request.

## Abbreviations

DMD: Duchenne muscular dystrophy
GC: Glucocorticoids
LC-MS/MS: Liquid chromatography mass spectrometry
QI: Quality improvement
Sal-T: Salivary testosterone
T: Testosterone
