# Supplementary material for: Measurement of salivary testosterone in adolescents and young men with Duchenne muscular dystrophy
Source: BMC Endocr Disord. 2021 Apr 10;21:63. doi: 10.1186/s12902-021-00727-4 (PMC8035731; doi:10.1186/s12902-021-00727-4)
Supplement: Supplementary file 1 — Additional file 1. Self-assessment questionnaire. [file 12902_2021_727_MOESM1_ESM.docx]

**Self-assessment questionnaire**

| Date of salivary sample collection |  | |
| --- | --- | --- |
| Time of salivary sample collection |  | |
| Your name |  | |
| Your date of birth |  | |
| Tanner self-assessment  Please look at these pictures and tick the one that best describes you | 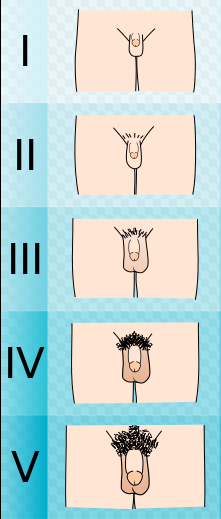 | ☐    ☐    ☐    ☐  ☐ |
| Current testosterone treatment? | YES ☐ NO ☐ | |
| Previous testosterone treatment? | YES ☐ NO ☐ | |
| If Yes, when was the last dose of testosterone given? | Approximate date : | |
| What form of testosterone? | Injection ☐ Tablet ☐ Gel ☐ Patch ☐ | |
| Do you take either prednisolone or deflazacort? | YES ☐ NO ☐ | |
| If yes, what dose? |  | |
